# Redefining Cardiac Biomarkers in Predicting Mortality of Inpatients With COVID-19

**DOI:** 10.1161/HYPERTENSIONAHA.120.15528

**Published:** 2020-07-14

**Authors:** Juan-Juan Qin, Xu Cheng, Feng Zhou, Fang Lei, Gauri Akolkar, Jingjing Cai, Xiao-Jing Zhang, Alice Blet, Jing Xie, Peng Zhang, Ye-Mao Liu, Zizhen Huang, Ling-Ping Zhao, Lijin Lin, Meng Xia, Ming-Ming Chen, Xiaohui Song, Liangjie Bai, Ze Chen, Xingyuan Zhang, Da Xiang, Jing Chen, Qingbo Xu, Xinliang Ma, Rhian M. Touyz, Chen Gao, Haitao Wang, Liming Liu, Weiming Mao, Pengcheng Luo, Youqin Yan, Ping Ye, Manhua Chen, Guohua Chen, Lihua Zhu, Zhi-Gang She, Xiaodong Huang, Yufeng Yuan, Bing-Hong Zhang, Yibin Wang, Peter P. Liu, Hongliang Li

**Affiliations:** 1 From the Department of Cardiology (J.-J.Q.), Zhongnan Hospital of Wuhan University, China; 2 Medical Science Research Center (F.Z., P.Z., H.L.), Zhongnan Hospital of Wuhan University, China; 3 Department of Hepatobiliary and Pancreatic Surgery (H.W., Y. Yuan), Zhongnan Hospital of Wuhan University, China; 4 Basic Medical School, Wuhan University, China (F.L., Z.H., X.S., Z.C., X.Z., H.L.); 5 Department of Cardiology (J.-J.Q., X.C., X.-J.Z., J.X., Y.-M.L., L.L., M.-M.C., L.Z., Z.-G.S., H.L.), Renmin Hospital of Wuhan University, China; 6 Department of Neonatology (B.-H.Z.), Renmin Hospital of Wuhan University, China; 7 Institute of Model Animal of Wuhan University, China (J.-J.Q., X.C., F.Z., F.L., X.-J.Z., P.Z., Y.-M.L., Z.H., L.-P.Z., L.L., M.X., M.-M.C., X.S., L.B., Z.C., X.Z., D.X., J. Chen, L.Z., Z.-G.S., H.L.); 8 Division of Cardiology, Department of Medicine, University of Ottawa Heart Institute, Ontario, Canada (G.A., A.B., P.P.L.); 9 Department of Cardiology, The Third Xiangya Hospital, Central South University, Changsha, China (J. Cai); 10 Centre for Clinic Pharmacology, The William Harvey Research Institute, Queen Mary University of London, United Kingdom (Q.X.); 11 Department of Emergency Medicine, Thomas Jefferson University, Philadelphia, Pennsylvania (X.M.); 12 Institute of Cardiovascular and Medical Sciences, BHF Glasgow Cardiovascular Research Centre, University of Glasgow, United Kingdom (R.M.T.); 13 Department of Anesthesiology, Cardiovascular Research Laboratories, David Geffen School of Medicine, University of California, Los Angeles (C.G., Y.W.); 14 Department of General Surgery, Ezhou Central Hospital, Wuhan, China (L.L.); 15 Department of General Surgery, Huanggang Central Hospital, Wuhan, China (W.M.); 16 Department of Urology (P.L.), Wuhan Third Hospital and Tongren Hospital of Wuhan University, China; 17 Department of Gastroenterology (X.H.), Wuhan Third Hospital and Tongren Hospital of Wuhan University, China; 18 Wuhan Seventh Hospital, China (Y. Yan); 19 Department of Cardiology, The Central Hospital of Wuhan, Tongji Medical College, Huazhong University of Science and Technology, China (P.Y., M.C.); 20 Department of Neurology, Wuhan First Hospital/Wuhan Hospital of Traditional Chinese and Western Medicine, Hubei, China (G.C.).

**Keywords:** biomarkers, heart diseases, heart injuries, mortality, prognosis

## Abstract

The prognostic power of circulating cardiac biomarkers, their utility, and pattern of release in coronavirus disease 2019 (COVID-19) patients have not been clearly defined. In this multicentered retrospective study, we enrolled 3219 patients with diagnosed COVID-19 admitted to 9 hospitals from December 31, 2019 to March 4, 2020, to estimate the associations and prognostic power of circulating cardiac injury markers with the poor outcomes of COVID-19. In the mixed-effects Cox model, after adjusting for age, sex, and comorbidities, the adjusted hazard ratio of 28-day mortality for hs-cTnI (high-sensitivity cardiac troponin I) was 7.12 ([95% CI, 4.60–11.03] *P*<0.001), (NT-pro)BNP (N-terminal pro-B-type natriuretic peptide or brain natriuretic peptide) was 5.11 ([95% CI, 3.50–7.47] *P*<0.001), CK (creatine phosphokinase)-MB was 4.86 ([95% CI, 3.33–7.09] *P*<0.001), MYO (myoglobin) was 4.50 ([95% CI, 3.18–6.36] *P*<0.001), and CK was 3.56 ([95% CI, 2.53–5.02] *P*<0.001). The cutoffs of those cardiac biomarkers for effective prognosis of 28-day mortality of COVID-19 were found to be much lower than for regular heart disease at about 19%–50% of the currently recommended thresholds. Patients with elevated cardiac injury markers above the newly established cutoffs were associated with significantly increased risk of COVID-19 death. In conclusion, cardiac biomarker elevations are significantly associated with 28-day death in patients with COVID-19. The prognostic cutoff values of these biomarkers might be much lower than the current reference standards. These findings can assist in better management of COVID-19 patients to improve outcomes. Importantly, the newly established cutoff levels of COVID-19–associated cardiac biomarkers may serve as useful criteria for the future prospective studies and clinical trials.

## Abstract 2

Supplemental Digital Content is available in the text.

The pandemic of coronavirus disease 2019 (COVID-19) caused by severe acute respiratory syndrome coronavirus 2 (SARS-CoV-2) infection has led to >11.8 million confirmed cases with >545 000 deaths worldwide by July 9, 2020.^1^ Patients with preexisting cardiovascular conditions are particularly at risk and have poor prognosis.^2^ With ACE2 (angiotensin-converting enzyme 2)—the binding and internalization receptor for SARS-COV2^3^—being highly expressed in the lung, heart, and the cardiovascular system, the heart is a target organ susceptible to viral and immune-mediated injury.^2,4,5^ Emerging data suggest that cardiac injury, manifested by cardiac biomarker elevation, is detected in a sizable of COVID-19 patients and is associated with adverse outcomes and increased mortality.^6,7^ However, how useful cardiac biomarkers are in COVID-19 prognosis and how to utilize these markers have not been well defined. This study aims to establish the role of cardiac-specific injury or stress biomarker and their precise association with COVID-19 mortality, to determine their use in prognostic evaluation of risk of death and to delineate the relationship of cardiac biomarker rise with other inflammatory markers in patients diagnosed with COVID-19.

## Methods

### Data, Materials, and Code Disclosure Statement

The data that support the findings of this study are available from the corresponding author upon reasonable request.

### Study Design and Participants

This retrospective multicenter cohort study enrolled patients diagnosed as COVID-19 and admitted to 9 hospitals in Hubei Province, China, from December 31, 2019 to March 4, 2020. COVID-19 diagnosis was confirmed by at least one or both criteria of chest computed tomography manifestations and reverse transcription–polymerase chain reaction according to the New Coronavirus Pneumonia Prevention and Control Program (fifth edition) published by the National Health Commission of China and World Health Organization interim guidance.^8,9^ The study design and procedures were approved by the central ethics committees, and all collaborating hospitals either approved the study protocol by local ethics committees or accepted the approval from the central ethics committee. Individual patient informed consent was waived by each ethics committee of participating hospitals.

For the analysis regarding the associations between increased cardiac injury markers and the risk of all-cause death of COVID-19, patients diagnosed with COVID-19 and having available high-sensitivity cardiac troponin I (hs-cTnI) or CK (creatine phosphokinase)-MB on admission were enrolled in this study. Participants aged <18 or >75 years, without complete electronic medical records due to transferring, or pregnant were excluded from the study. The primary end point was 28-day all-cause mortality, and the time of hospital admission was designed as day 0. The increase of serum myocardial marker levels and other biochemical markers was defined as above the upper limit of normal (ULN) according to local hospital criteria. Heart injury status was defined as a serum level of hs-cTnI or CK-MB above the ULN. Due to variations in the examining instruments, laboratory techniques, and regents, different ULNs were applied at different hospital sites. To guarantee the accuracy and broad-based applicability, the levels of cardiac injury biomarkers were normalized and analyzed as relative values to their local ULNs.

### Data Collection

The demographic information, preexisting comorbidities, clinical characteristics, chest computed tomography radiological data, laboratory data, and clinical outcomes were collected from electronic medical record data. Clinical characteristics including heart rate, breath rate, fever, cough, and dyspnea at the time of admission were analyzed. Laboratory measurements including neutrophil count, CRP (C-reactive protein), cardiac biomarkers (hs-cTnI, CK-MB, NT-proBNP [N-terminal pro-B-type natriuretic peptide] or BNP [brain natriuretic peptide], MYO [myoglobin], and CK), and inflammatory cytokine IL (interleukin)-6 at admission and during hospitalization were extracted and analyzed. The unilateral and bilateral lung lesion status was obtained from computed tomography images. Personal identifiers of included participants were first anonymized and replaced by study ID before data collection. All the clinical data were individually reviewed and verified by a team of experienced physicians blinded to patient identification.

### Statistical Analysis

Data in this study were analyzed using R-3.6.3 (R Foundation for Statistical Computing, Vienna, Austria) and SPSS Statistics (version 23.0; IBM, Armonk, NY). Distribution of myocardial marker levels (normalized to ULN) by age and sex was displayed using kernel density estimation. Dynamic change of myocardial marker levels in the cumulative proportions of patients was created and smoothed by locally weighted regression and smoothing scatterplots. The Akaike information criterion scores and *P* values were calculated by the univariate logistic regression analysis to estimate the associations of variables with 28-day all-cause death of COVID-19 and were examined by the multivariate Cox analysis to evaluate the quality of the model. A mixed-effects Cox model analysis was conducted to determine the association between baseline myocardial marker levels and the risk of the primary end point of COVID-19 by treating the site as a random effect. Hazard ratios (HRs) and 95% confidence interval (CI) were calculated in the mixed-effects model. Multivariable adjustment included age, sex, and preexisting comorbid conditions (diabetes mellitus, hypertension, coronary heart disease, and cerebrovascular disease). Kaplan-Meier method was applied to compare the cumulative survival between participants with normal versus increased myocardial marker levels and between those with myocardial marker levels under or above cutoffs. The receiver operating characteristic analysis was conducted to assess the overall performances of increased levels of myocardial injury parameters for identification of the risk of mortality in patients with COVID-19. The area under the receiver operating characteristic curve (AUC) was computed for evaluating the performance of each marker level. A 2-sided α-error <0.05 was considered statistically significant.

## Results

### Participants and Clinical Characteristics

A total of 7106 participants diagnosed with COVID-19 admitted to 9 hospitals in Hubei Province, China, were recruited initially in this study. Of these, 277 cases were excluded due to incomplete electronic medical records during interhospital transfer, 767 patients were excluded due to age limitation of the study design (>75 or <18 years), and 29 cases were excluded due to pregnancy. In the remaining patients, 3219 participants had measured CK-MB or hs-cTnI levels, while 2814 cases were not. The clinical symptoms, basic comorbidities, laboratory data, and outcomes of those patients are shown in Table 1 and Table S1 in the Data Supplement. Compared with patients without cardiac injury biomarker measurement, patients with biomarker values were older (median age, 57 [interquartile range, 45–66] versus 54 [interquartile range, 42–64] years) and had higher percentages of preexisting comorbidities and more severe symptoms (Tables S1 and S2). The frequencies for increase of hs-cTnI, CK-MB, (NT-pro)BNP (BNP or NT-proBNP), CK, and MYO were 6.5%, 5.1%, 12.9%, 12.1%, and 12.0% on admission among patients with available cardiac biomarker values, respectively. The exact values and normal ranges of laboratory examinations in patients with and without myocardial biomarker measurements are shown in Tables S2 and S3.

**Table 1.**
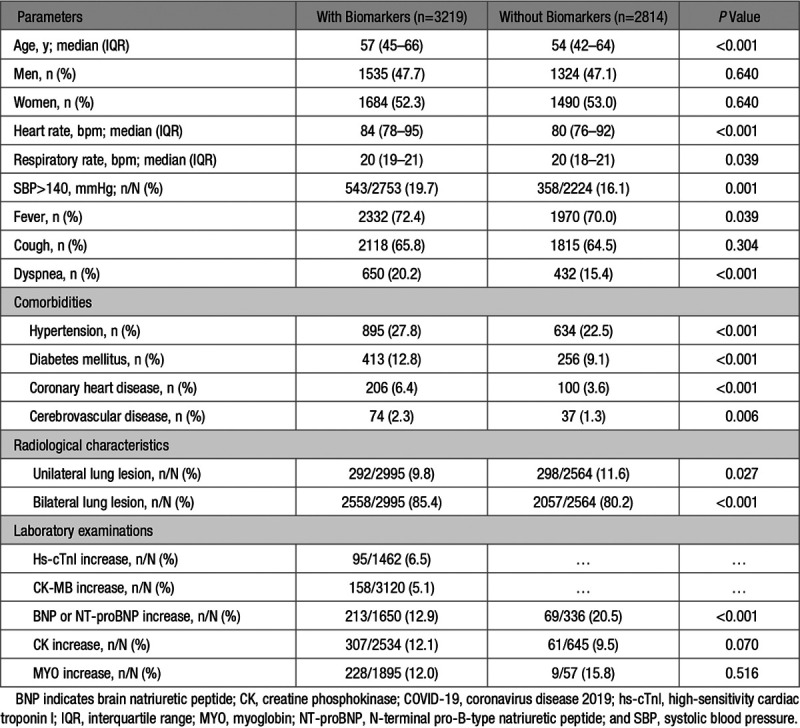
Baseline Characteristics of Patients With COVID-19

The distribution of age- and sex-specific myocardial biomarker profiles relative to those of ULN at presentation were analyzed and presented as kernel density plots (Figure S1). The age brackets were divided into 18 to 44, 45 to 59, and 60 to 75 years. The distributions of CK-MB, CK, and MYO showed higher levels in men than in women among all 3 age brackets, while hs-cTnI and (NT-pro)BNP had comparable distributions between male and female patients at the time of admission.

### Association of Cardiac Biomarkers With 28-Day Mortality of COVID-19

To evaluate associations between outcomes of COVID-19 and covariates including cardiac injury markers, we performed univariate logistic regression analysis where the Akaike Information Criterion and *P* values were calculated. Notably, all 5 myocardial biomarkers (hs-cTnI, CK-MB, (NT-pro)BNP, CK, and MYO) were significantly associated with 28-day all-cause death of COVID-19 (Table S4).

To evaluate the association of each myocardial marker elevation above laboratory-defined ULN with the primary end point of all-cause mortality at 28 days post-admission, we conducted a mixed-effects Cox model analysis using the patient’s hospital site as a random effect. Multivariables adjustments included age, sex, and coexisting comorbidities (hypertension, diabetes mellitus, coronary heart disease, and cerebrovascular disease). Adjusted HRs for associations of 28-day all-cause mortality of COVID-19 with abnormally elevated hs-cTnI, CK-MB, NT-proBNP, CK, and MYO were shown in Table 2, with 95% CI. Elevation of hs-cTnI exhibited the highest adjusted HR of 7.12 ([95% CI, 4.60–11.03] *P*<0.001), followed by (NT-pro)BNP of 5.11 ([95% CI, 3.50–7.47] *P*<0.001), CK-MB of 4.86 ([95% CI, 3.33–7.09] *P*<0.001), and MYO of 4.50 ([95% CI, 3.18–6.36] *P*<0.001). Total CK—a much less specific cardiac biomarker—had an adjusted HR of 3.56 ([95% CI, 2.53–5.02] *P*<0.001).

**Table 2.**
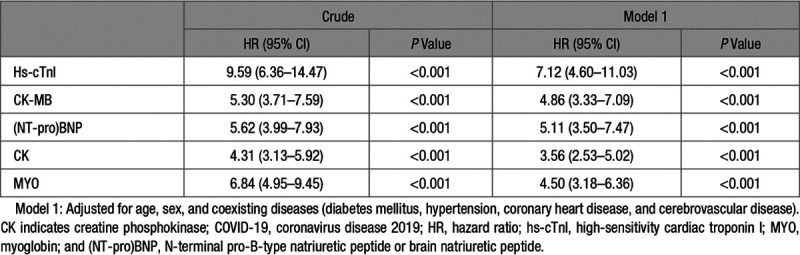
Association of Increased Cardiac Injury Markers Above the Upper Limit of Normal With 28-d All-Cause Mortality of COVID-19

**Figure 1.**
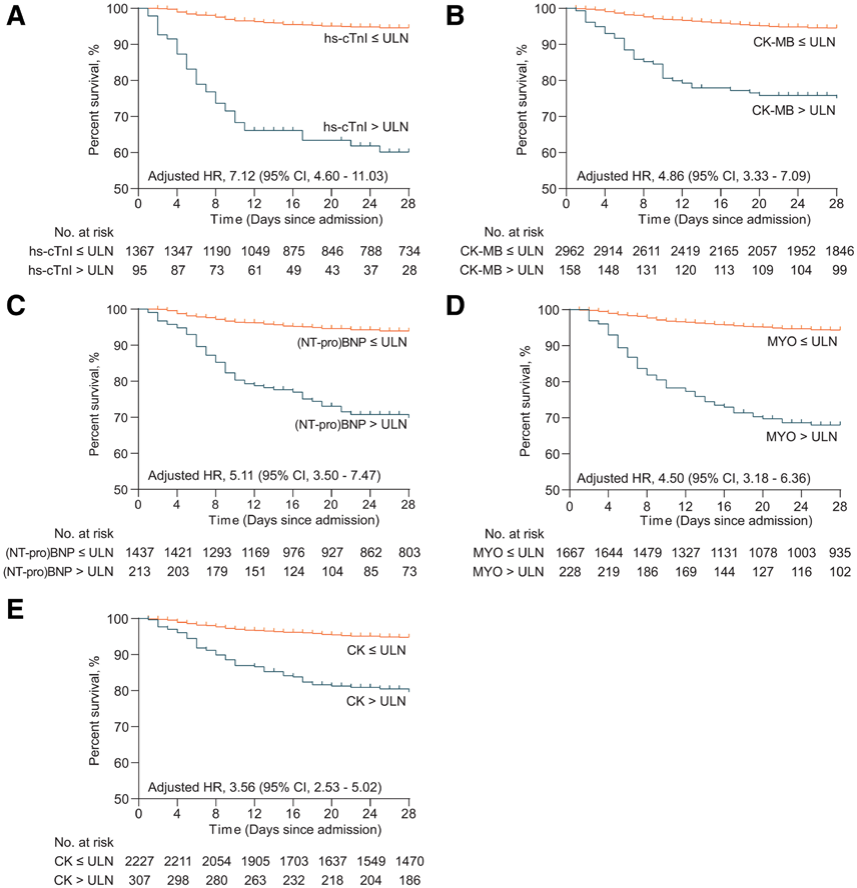
Survival profiles of COVID-19 patients stratified according to cardiac biomarkers. Kaplan-Meier curves showing cumulative survival of coronavirus disease 2019 (COVID-19) patients with increased or normal levels of high-sensitivity cardiac troponin I (hs-cTnI; **A**), CK (creatine phosphokinase)-MB (**B**), (NT-pro)BNP (N-terminal pro-B-type natriuretic peptide or brain natriuretic peptide; **C**), MYO (myoglobin; **D**), and CK (**E**) during a 28-d follow-up. The increase of myocardial marker represents level above the upper limit of normal (ULN). HR indicates hazard ratio.

Kaplan-Meier curves illustrated that patients with increased hs-cTnI, CK-MB, (NT-pro)BNP, CK, and MYO had significantly decreased survival rate compared with those with normal levels (Figure 1). The Kaplan-Meier curves demonstrated an early separation of the mortality curves between patients showing elevated admission biomarker values above ULN, versus those having normal values, underscoring the rapid onset of death among the high-risk group of COVID-19 patients. This was particularly prominent for cardiac-specific biomarkers, such as hs-cTnI, CK-MB, and (NT-pro)BNP.

Compared with patients without available myocardial biomarkers, patients with measured cardiac injury markers had higher incidences of 28-day all-cause death and the occurrences of Acute Respiratory Distress Syndrome, heart failure, disseminated intravascular coagulation (DIC), sepsis, or multiorgan failure and acute renal failure (Table S5). However, there were no significant differences in the main causes of death between these 2 subcohorts (Table S6). These data suggested that heart injury markers might sensitively respond to SARS-CoV-2 infection or infection-related outcomes.

### Prognostic Performance of Cardiac Injury Biomarkers in Predicting 28-Day All-Cause Mortality of COVID-19

To compare the relative accuracy, sensitivity, specificity, and positive and negative predictive values of each biomarker based on laboratory-defined ULN, the prognostic performance of each marker was analyzed. The receiver operating characteristic curve was used to demonstrate the ability of each cardiac biomarker in discrimination of high risk of COVID-19 mortality, which was quantitated as AUC. Increase of MYO—an early release biomarker of cardiac injury—showed the highest overall performance (AUC, 0.83 [95% CI, 0.80–0.86]) to predict the risk of COVID-19 mortality, followed by (NT-pro)BNP (AUC, 0.81 [95% CI, 0.78–0.85]), hs-cTnI (AUC, 0.78 [95% CI, 0.73–0.84]), and CK-MB (AUC, 0.71 [95% CI, 0.67–0.75]). CK had the lowest performance (AUC, 0.67 [95% CI, 0.62–0.72]; Table 3; Figure S2). It is useful to note that the overall negative predictive values are uniformly high for any of these biomarkers, at over 96% for all of those evaluated.

The standard laboratory cutoff value of ULN is usually defined as the 99th upper percentile of biomarker distribution in the normal population. However, our data indicated that the operational cutoff based on the biomarker distribution and receiver operating characteristic performance curve should be redefined for each of the biomarkers to enhance the prognostic sensitivity of the biomarkers. Indeed, the operational cutoff values of hs-cTnI, CK-MB, (NT-pro)BNP, CK, and MYO for COVID-19 mortality prediction were markedly lower, calculated at 49.0%, 49.1%, 18.9%, 44.8%, and 49.8% of their respective ULNs. Notably, using these cutoff values of each myocardial marker in this risk prediction model improved the balanced accuracy versus using the currently recommended ULN values (0.77 versus 0.66 for hs-cTnI, 0.66 versus 0.59 for CK-MB, 0.75 versus 0.66 for (NT-pro)BNP, 0.75 versus 0.69 for MYO, and 0.64 versus 0.63 for CK) than using the current ULN values defined in the laboratory (Table 3; Table S7).

Accordingly, patients with biomarker levels above each recalibrated cutoff were at a significantly higher risk of 28-day all-cause mortality of COVID-19, compared with those under the cutoff points (Figure S3). After adjusted for age, sex, and comorbidities in the mixed-effects Cox model and treated hospital site as a random effect, patients with elevated myocardial biomarkers over the new cutoffs were at a significantly higher risk of mortality than those having biomarker levels under cutoff values with adjusted HR of 10.68 ([95% CI, 6.87–16.59] *P*<0.001) for hs-cTnI, 3.63 ([95% CI, 2.60–5.06] *P*<0.001) for CK-MB, 12.01([95% CI, 6.39–22.58] *P*<0.001) for (NT-pro)BNP, 2.7 ([95% CI, 1.92–3.81] *P*<0.001) for CK, and 5.00 ([95% CI, 3.37–7.42] *P*<0.001) for MYO (Table S8).

To address the potential selection bias, we further adjusted for the imbalanced variables related to disease severity (CRP increase, neutrophil count increase, lymphocyte count decrease, d-dimer increase, and Spo_2_ <95%) between patients with or without increased heart injury markers above cutoff values in the mixed-effects Cox model. The results showed that the increased hs-cTnI (adjusted HR, 4.74 [95% CI, 3.05–7.35]; *P*<0.001), CK-MB (adjusted HR, 2.17 [95% CI, 1.56–3.01]; *P*<0.001), CK (adjusted HR, 1.80 [95% CI, 1.28–2.53]; *P*<0.001), (NT-pro)BNP (adjusted HR, 5.67 [95% CI, 2.97–10.82]; *P*<0.001), and MYO (adjusted HR, 2.74 [95% CI, 1.82–4.13]; *P*<0.001) were still significantly associated with higher risk of all-cause mortality of COVID-19 (Table S8). The Akaike information criterion values of the crude and adjusted analysis model were shown in Table S9, among which the model adjusted for age, sex, and comorbidities had relatively low Akaike information criterion values for each myocardial marker.

We further performed a subgroup sensitivity analysis using patients in site 1, site 2, site 3, and site 6, where a relatively high percentage of patients had CK-MB or hs-cTnI measured (70.0% in site 1 and site 2, 82.8% in site 3, and 68.3% in site 6). In the mixed-effects Cox model, after adjusting for age, sex, and coexisting comorbidities, the significant associations between increased cardiac injury biomarkers and a higher risk of COVID-19 mortality were still maintained with adjusted HR of 4.42 ([95% CI, 2.74–7.13] *P*<0.001) for hs-cTnI, 2.98 ([95% CI, 1.94–4.59] *P*<0.001) for CK-MB, 5.46 ([95% CI, 2.55–11.73] *P*<0.001) for (NT-pro)BNP, 1.57 ([95% CI, 1.00–2.45] *P*=0.048) for CK, and 2.92 ([95% CI, 1.76–4.85] *P*<0.001) for MYO.

In another sensitivity analysis, we excluded patients with acute myocardial infarction to mitigate heart disease history-related potential confounders. After adjusting for age, sex, and comorbidities, the increased CK-MB (adjusted HR, 4.39 [95% CI, 2.94–6.56]; *P*<0.001), hs-cTnI (adjusted HR, 6.95 [95% CI, 4.48–10.8]; *P*<0.001), CK (adjusted HR, 3.45 [95% CI, 2.43–4.90]; *P*<0.001), (NT-pro)BNP (adjusted HR, 4.88 [95% CI, 3.31–7.20]; *P*<0.001), and MYO (adjusted HR, 4.20 [95% CI, 2.94–6.00]; *P*<0.001) remained to be significantly associated with higher risk of all-cause mortality of COVID-19 in this subcohort.

**Figure 2.**
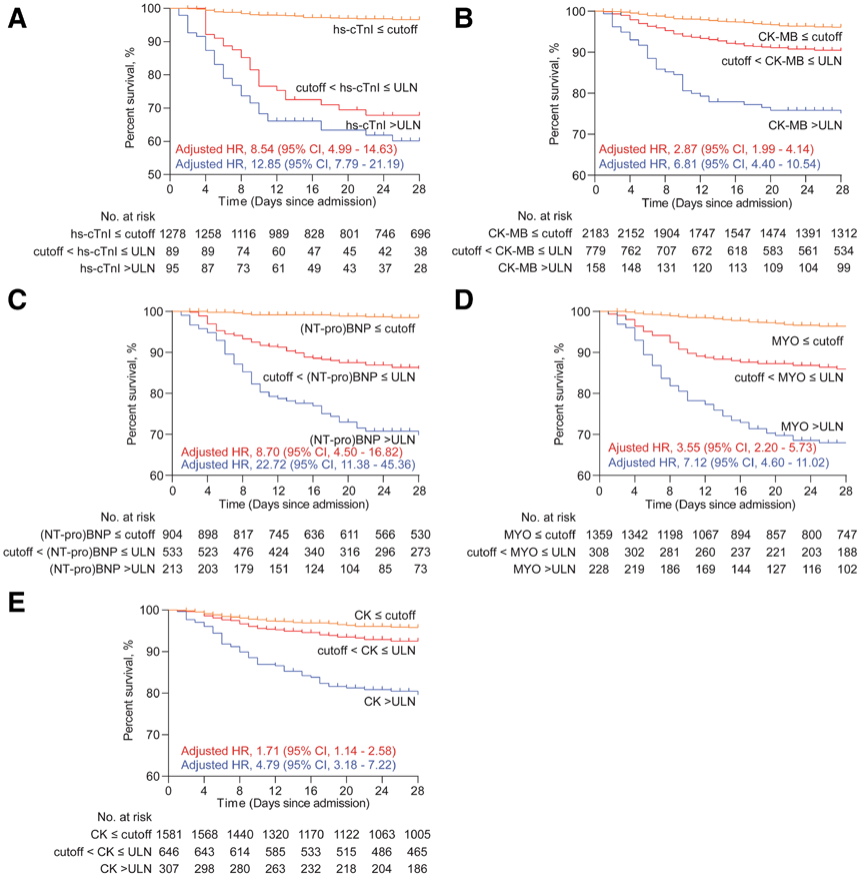
The percentage survival of patients with cardiac biomarker levels under cutoffs, between cutoffs and upper limits of normal (ULNs), and above ULNs of high-sensitivity cardiac troponin I (hs-cTnI; **A**), CK (creatine phosphokinase)-MB; **B**), (NT-pro)BNP (N-terminal pro-B-type natriuretic peptide or brain natriuretic peptide); **C**), MYO (myoglobin; **D**), and CK (**E**). HR indicates hazard ratio.

More importantly, the patients with cardiac biomarker levels ranging from the newly established cutoffs to laboratory reference ULNs still showed significantly lower percentage survival than those with marker levels below the newly established cutoff values (Figure 2). The adjusted HRs for 28-day all-cause death of patients with cardiac biomarker between cutoffs and ULNs were 8.54 ([95% CI, 4.99–14.63] *P*<0.001) for hs-cTnI, 2.87 ([95% CI, 1.99–4.14] *P*<0.001) for CK-MB, 8.70 ([95% CI, 4.50–16.82] *P*<0.001) for (NT-pro)BNP, 3.55 ([95% CI, 2.20–5.73] *P*<0.001) for MYO, and 1.71 ([95% CI, 1.14–2.58] *P*=0.009) for CK compared with those with marker levels below cutoffs (Table 4). This means patients with even borderline levels of biomarker (between ULN and the newly established cutoffs) as defined by our current study might still have a higher risk of 28-day mortality.

**Table 3.**
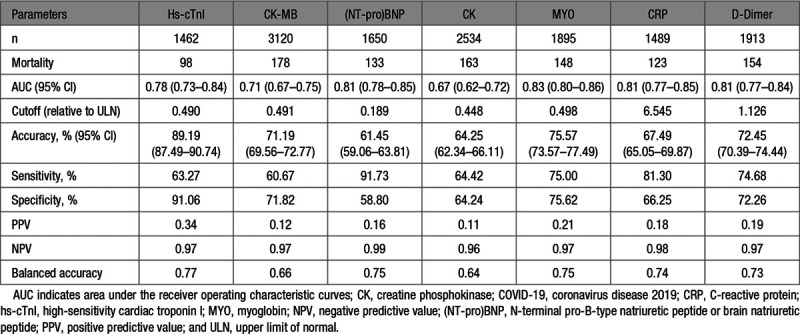
Overall Performance of Cardiac Biomarkers for Predicting COVID-19 Mortality According to Receiver Operating Characteristic Curves

**Table 4.**
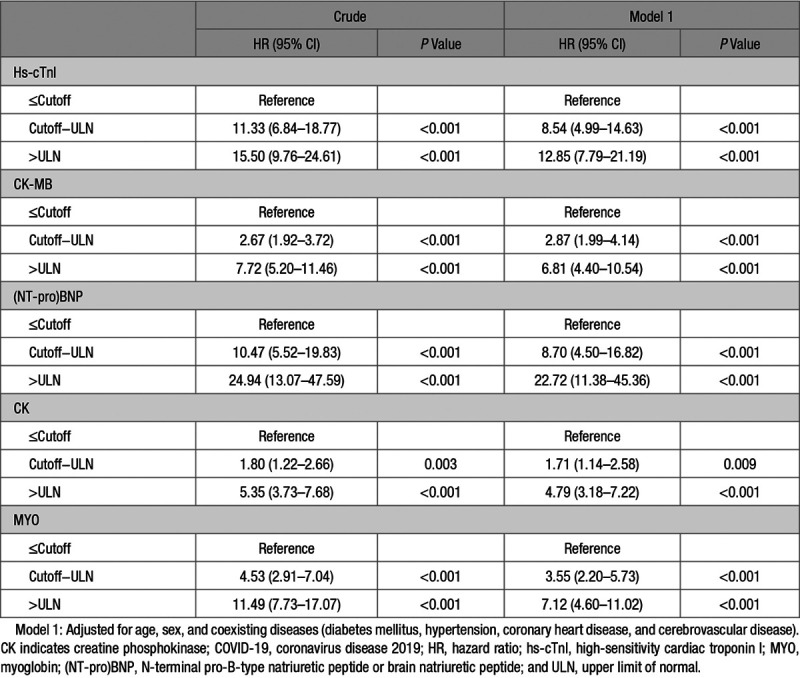
Association of Cardiac Injury Markers With 28-d All-Cause Mortality of COVID-19 in Patients Divided by Cutoffs and Upper Limit of Normal

Overall, we found the number of patients showing biomarkers above the newly established cutoff values but below the laboratory-defined ULNs at admission were 89 for hs-cTnI, 779 for CK-MB, 533 for (NT-pro)BNP, 308 for MYO, and 646 for CK (Figure 2). These data revealed that the prevalence of cardiac injury might be substantially underestimated by the current laboratory-defined ULN values.

### Trajectory Patterns of Cardiac Biomarkers and Inflammatory Factor Elevation in Patients With COVID-19

To further demonstrate the mechanistic cause of cardiac injury in COVID-19 patients, we determined the temporal relationship of cardiac biomarker elevation with that of the inflammatory markers over time, we analyzed the cumulative proportions of patients with increased cardiac biomarkers in association with inflammatory factors of CRP, neutrophil count, and IL-6 elevation during the entire study period. This was based on the laboratory-defined ULN and analyzed from time of symptom onset (day 0) to the end of follow-up, as illustrated in Figure S4.

Among the patients without signs of heart injury (defined by having elevated hs-cTnI or CK-MB above their ULN), the increases of inflammatory markers were lower and slower than those with heart injury (Figure S4A). In patients showing heart injury during the entire hospitalization, neutrophil percentage and CRP were rapidly and simultaneously increased after disease onset, immediately followed by the increases of CK-MB, MYO, and hs-cTnI. In contrast, the significant elevation of IL-6 occurred only after the increases of these myocardial markers and was highly elevated mainly in patients with evidence of cardiac injury (Figure S4A).

We further divided patients into poor outcome and favorable outcome groups according to the occurrence of death, intensive care unit treatment, and mechanical ventilation from admission to the end of follow-up, where patients with any one of the above events were classified as in poor outcome and patients without any of the above events were in favorable outcome group. The temporal patterns of myocardial biomarkers and inflammatory factors were cosegregated between these 2 groups (Figure S4B). Compared with patients with favorable outcomes, the increases of neutrophil percentage and CRP showed sharper slope in patients with poor outcome. The proportion of patients with increased hs-cTnI was significantly higher in the poor outcome than in the favorable outcome group. Patients with minimum levels of (NT-pro)BNP had overall a much more favorable outcome. Notably, the elevation of serum IL-6 level was highly prognostic of poor outcome and followed slightly behind cardiac biomarker elevation such as hs-cTnI in patients with poor outcome. In contrast, the increase of IL-6 was significantly delayed and blunted in amplitude in those with favorable outcome (Figure S4B). Furthermore, the magnitudes of the inductions in both myocardial markers and inflammatory factors were higher in the poor outcome patients than in the ones with favorable outcome (Figure S5).

The increased inflammatory marker (CRP) and coagulation marker (d-dimer) levels were also significantly associated with an increased risk of 28-day all-cause mortality of COVID-19 and had interactive effects with cardiac injury markers in predicting the poor outcomes of COVID-19 (Table 3; Table S10).

## Discussion

This retrospective study is a comprehensive evaluation of serum cardiac biomarkers with respect to 28-day all-cause mortality of COVID-19. We demonstrated that elevations of biomarkers such as hs-cTnI, CK-MB, (NT-pro)BNP, or MYO based on reference laboratory normal cutoff values were highly prognostic of 28-day all-cause mortality, including deaths occurring soon after admission. However, standard cutoff values currently used for diagnosis likely underestimated the true extent of cardiac injury. The newly established cutoffs in our study for COVID-19 prognosis were much lower than the currently accepted laboratory cutoff thresholds (by about 50%). Dynamic pattern of cardiac biomarker elevation showed their onset coincided with CRP and neutrophil elevation but preceded the elevation of IL-6.

These findings are consistent with earlier reports that cardiac injury biomarkers are associated with an increased risk of COVID-19 mortality.^7,10^ Elevated levels of cardiac troponin I and (NT-pro)BNP, CK-MB, and MYO are also associated with more severe symptoms and disease progression.^11–14^ Our larger sample size helped to better define and to improve the performance characteristics of these biomarkers for COVID-19, which manifest with widely divergent outcomes from full recovery to rapid death. The cardiac-specific biomarkers, such as hs-cTnI, CK-MB, and (NT-pro)BNP, generally have better performance than nonspecific markers such as CK. The lower levels of newly established cutoff values of cardiac injury markers further suggest that the currently used laboratory ULNs for these markers, based on the 99 percentiles of distribution in a normal population, might significantly underestimate the extent of cardiac injury associated with COVID-19.

The ULNs of cardiac injury markers are primarily established and extensively recognized for diagnosis of the severity of the cardiac injury and thus would have the satisfactory performance for predicting the prognosis of patients with cardiovascular diseases. Therefore, it is interesting that outcomes from COVID-19—a disease associated primarily with the lung—are also well predicted by cardiac biomarkers. While the inherent limitation of a retrospective study makes it impossible to demonstrate the causal effect of increased cardiac injury markers with COVID-19 death, the performance of these markers for predicting COVID-19 prognosis may indicate pathological impact of SARS-CoV-2 infection or the infection-related pathogenesis on myocardium. In consistence with this hypothesis, Angeli et al found a wide spectrum of ECG abnormalities during hospitalization, including persistent ST-T changes, atrial fibrillation, and brady-tachy syndrome, in a prospective study containing 50 inpatients with COVID-19. Among those with abnormal electrocardiogram performance, 38% of them were recorded with abnormal serum levels of hs-cTnI.^15^

Heart injury may likely involve multiple mechanisms, including primary injury from SARS-COV-2 infection and indirect injury from the imbalanced immune response with dominant macrophage and neutrophil effects. This can be augmented further by systemic or cardiac hypoxemia and microangiopathy and microthormbi.^2^ ACE2—the functional receptor and a portal of entry for both SARS-COV and SARS-COV-2—is highly expressed in the heart and vasculature.^3,16,17^ Previous studies indicated that SARS-COV is detectable in the heart of infected patients^18^ and ACE2 expression is downregulated after coronavirus infection, leading to excessive activation of the renin-angiotensin-aldosterone system, which can further exacerbate myocardial injury.^19,20^ The link between SARS-COV-2 and ACE2 provides a possible mechanism that SARS-COV-2 binds to ACE2 expressed on cardiomyocytes/fibroblasts and induces ACE2 downregulation and renin-angiotensin-aldosterone system dysfunction, which in turn leads to heart dysfunction and pneumonia progression. Our most recent study indicated that inpatients using ACE inhibitors or angiotensin II receptor blockers are at significantly lower risk of 28-day all-cause death compared with nonusers in patients with hypertension hospitalized with COVID-19.^21^ Autopsy analysis of hearts from COVID-19 patients suggested a potential involvement of systematic inflammation in heart injury.^22^ However, there are, so far, no data directly demonstrating the presence of SARS-CoV-2 within myocardial tissue or the direct influence of renin-angiotensin-aldosterone system inhibitors on heart injury in COVID-19 patients.

There are several limitations of our study. First, the inherent limitations of retrospective studies make it impossible to determine whether cardiac injury is the causal effect on the prognosis of COVID-19. Second, the cutoffs of cardiac injury markers in predicting COVID-19 outcomes were established based on a Chinese population, which will require further external validations from other independent cohorts. Third, in the urgent circumstances during the COVID-19 pandemic, not all markers and examinations (eg, electrocardiogram, ultrasound computed tomography and magnetic resonance imaging) related to heart injury were fully performed and collected during the entire hospitalization period from all participants. The incomplete data collection in multiple sites might lead to confounding effects on the magnitude of the association between cardiac injury markers and COVID-19 prognosis and the predicting performance of those markers for COVID-19 death. Fourth, because of the limited sample size, it is unknown whether those myocardial markers are also effective in predicting the outcomes of COVID-19 in patients with different complications or comorbidities. Fifth, as all participants involved in this study were inpatients, findings in this study might not be fully applicable to all individuals in the general population infected with SARS-COV-2, such as outpatients in isolation sites or community. Sixth, there might be selection bias of the study subjects because myocardial markers may be examined more frequently in patients with suspected heart injury or with signs of cardiovascular diseases than in the general patient population. The imbalanced history of heart failure, the time point of admission, and the availability of medical resources might also introduce confounding factors into our conclusion. Thus, associations between cardiac biomarkers and COVID-19 mortality and the exact cutoffs of those markers for COVID-19 prognosis with adequate sensitivity and specificity in the whole patient population need to be further investigated in large-scale general population and in the rigorously designed prospective studies and randomized controlled trials.

In conclusion, the abnormal cardiac biomarker pattern in COVID-19 patients was significantly associated with increased mortality risk, and the newly established COVID-19 prognostic cutoff values of hs-cTnI, CK-MB, (NT-pro)BNP, CK, and MYO were found to be much lower (≈50%) than reference upper normal limits for the general population. It is clinically meaningful that the fluctuating levels of myocardial biomarkers should be intensively monitored, and patients with elevated levels of those biomarkers should be intervened timely to improve the prognosis of COVID-19. Our findings support additional prospective studies and randomized controlled clinical trials to accurately validate the risk thresholds and exact impact of myocardial injury for individuals with COVID-19.

## Perspectives

To properly evaluate patients with COVID-19 at admission, the cutoff threshold of abnormality for hs-cTnI, CK-MB, (NT-pro)BNP, CK, and MYO at admission should be lower than the currently recommended laboratory range. Using standard reference laboratory cutoffs might underestimate the extent of cardiac injury. Measurement of cardiac-specific biomarkers and proper interpretation on admission can help to identify COVID-19 patients with a high-risk trajectory. The elevations can help to provide references for the management, monitoring, and enrollment for prospective studies and randomized controlled clinical trials.

## Acknowledgments

J.-J. Qin, X. Cheng, F. Zhou, and F. Lei designed the study, collected and analyzed data, and wrote the manuscript. G. Akolkar, J. Cai, X.-J. Zhang, and A. Blet designed the study and wrote the manuscript. J. Xie, P. Zhang, Z. Huang, L.-P. Zhao, L. Lin, M. Xia, M.-M. Chen, X. Song, L. Bai, Z. Chen, X. Zhang, and D. Xiang collected and reviewed clinical, laboratory, and radiological data. Y.-M. Liu and J. Chen performed the statistical analysis. Q. Xu, X. Ma, R.M. Touyz, C. Gao, and Z.-G. She edited the manuscript and provided valuable suggestions for the study design and data analysis. H. Wang, L. Liu, W. Mao, P. Luo, Y. Yan, P. Ye, M. Chen, G. Chen, L. Zhu, X. Huang, B.-H. Zhang, and Y. Yan reviewed, interpreted, and checked clinical data. Y. Wang, P.P. Liu, and H. Li contributed equally, designed the project, edited the manuscript, and supervised the study. All authors have approved the final version of this article.

## Sources of Funding

None.

## Disclosures

None.

## Supplemental Materials

Online Tables I–X

Online Figures I–V

## Supplementary Material



## Novelty and Significance

### What Is New?

- Heart injury is one of the major complications in patients with coronavirus disease 2019 (COVID-19).
- However, the prognostic power of cardiac biomarkers, their utility, and pattern of release in COVID-19 patients have not been clearly defined.

### What Is Relevant?

- This multicentered retrospective cohort study involving 3219 patients demonstrated that hazard ratios for admission cardiac biomarkers such as high-sensitivity cardiac troponin I, CK (creatine phosphokinase)-MB, or (NT-pro)BNP (N-terminal pro-B-type natriuretic peptide or brain natriuretic peptide) for 28-d mortality is high at between 4 and 7 folds.
- The established cutoff values of each cardiac biomarker to predict the risk of COVID-19 mortality are actually much lower than standard reference laboratory cutoffs by being about 50% lower.
- Trajectory analysis showed cardiac injury biomarkers are elevated early in course of disease, being parallel to CRP (C-reactive protein) and neutrophil rise, but precedes rise in IL (interleukin)-6 and predicts mortality, ventilator use, and intensive care unit admission.

### Summary

To properly prognosticate patients with COVID-19 at admission, the cutoff threshold of abnormality for high-sensitivity cardiac troponin I, CK-MB, (NT-pro)BNP, CK, and MYO (myoglobin) was analyzed as about 50% lower for most of the markers. Using standard reference laboratory cutoffs might underestimate the extent of cardiac injury. Admission cardiac biomarkers are sensitively prognostic for impending mortality in COVID-19. Their elevation tracks CRP and neutrophil rise but precedes IL-6 elevation. Measurement of cardiac-specific biomarkers and proper interpretation on admission can help to identify COVID-19 patients with a high-risk trajectory. The elevations provide support to guide management, monitoring, and enrollment into prospective studies and randomized controlled clinical trials.
