# Supplementary material for: Redefining Cardiac Biomarkers in Predicting Mortality of Inpatients With COVID-19
Source: Hypertension. 2020 Jul 14;76(4):1104–12. doi: 10.1161/HYPERTENSIONAHA.120.15528 (PMC7375179; doi:10.1161/HYPERTENSIONAHA.120.15528)
Supplement: Supplementary file 1 [file hyp-76-1104-s001.pdf]

Data Supplement

Redefining cardiac biomarkers in predicting mortality and adverse outcomes of inpatients with COVID-19

Juan-Juan Qin*, MD[1-3]; Xu Cheng*, PhD[2,3]; Feng Zhou*, PhD[1,4]; Fang Lei*, MS[1,3]; Gauri Akolkar, MD[5]; Jingjing Cai, MD[6]; Xiao-Jing Zhang, PhD[2,3]; Alice Blet, MD[5]; Jing Xie, MD[2]; Peng Zhang, PhD[2,4]; Ye-Mao Liu, PhD[2,3]; Zizhen Huang, MS[1,3]; Ling-Ping Zhao, MS[3]; Lijin Lin, MS[2,3]; Meng Xia, MS[3]; Ming-Ming Chen, MS[2,3]; Xiaohui Song, MS[1,3]; Liangjie Bai, MS[3]; Ze Chen, MS[1,3]; Xingyuan Zhang, MS[1,3]; Da Xiang, MS[3]; Jing Chen, DrPh, MS[3]; Qingbo Xu, PhD [7]; Xinliang Ma, PhD [8]; Rhian M. Touyz, MD[9]; Chen Gao, MD[10], Haitao Wang, MD[11]; Liming Liu, MD[12]; Weiming Mao, MD[13]; Pengcheng Luo, MD[14]; Youqin Yan, MD[15]; Ping Ye, MD[16]; Manhua Chen, MD[16]; Guohua Chen, MD[17]; Lihua Zhu, MD[2,3]; Zhi-Gang She, MD[2,3]; Xiaodong Huang, MD[18]; Yufeng Yuan, MD[11]; Bing-Hong Zhang, MD[19]; Yibin Wang, PhD[10]; Peter P. Liu, PhD[5]; Hongliang Li, PhD, MD[1-4]

1. Department of Cardiology, Zhongnan Hospital of Wuhan University; Basic Medical School, Wuhan University, Wuhan, China;
2. Department of Cardiology, Renmin Hospital of Wuhan University, Wuhan, China;
3. Institute of Model Animal of Wuhan University, Wuhan, China;
4. Medical Science Research Center, Zhongnan Hospital of Wuhan University, Wuhan, China;
5. Division of Cardiology, Department of Medicine, University of Ottawa Heart Institute, Ottawa, Ontario, Canada
6. Department of Cardiology, The Third Xiangya Hospital, Central South University, Changsha, China;
7. Centre for Clinic Pharmacology, The William Harvey Research Institute, Queen Mary University of London, UK;
8. Department of Emergency Medicine, Thomas Jefferson University, Philadelphia, Pennsylvania, PA 19004, USA
9. Institute of Cardiovascular and Medical Sciences, BHF Glasgow Cardiovascular Research Centre, University of Glasgow, Glasgow, UK;
10. Department of Anesthesiology, Cardiovascular Research Laboratories, David Geffen School of Medicine, University of California, Los Angeles, USA;
11. Department of Hepatobiliary and Pancreatic Surgery, Zhongnan Hospital of Wuhan University, Wuhan;
12. Department of general surgery, Ezhou Central Hospital, Wuhan, China;

13. Department of general surgery, Huanggang Central Hospital, Wuhan, China;

14. Department of Urology, Wuhan Third Hospital & Tongren Hospital of Wuhan University, Wuhan, China;

15. Infections Department, Wuhan Seventh Hospital, Wuhan, China;

16. Department of Cardiology, The Central Hospital of Wuhan, Tongji Medical College, Huazhong University of Science and Technology, Wuhan, China;

17. Department of Neurology, Wuhan First Hospital / Wuhan Hospital of Traditional Chinese and Western Medicine, Wuhan, Hubei, China.

18. Department of Gastroenterology, Wuhan Third Hospital & Tongren Hospital of Wuhan University, Wuhan, China;

19. Departments of Neonatology, Renmin Hospital of Wuhan University, Wuhan, China;

*These authors contributed equally.

Correspondence to:

Prof **Peter P. Liu**
Division of Cardiology, Department of Medicine, University of Ottawa Heart Institute, 40 Ruskin Street, Ottawa, Ontario K1Y 4W7, Canada; Tel: 613-798-5555; Email: peter.liu@utoronto.ca OR pliu@ottawaheart.ca

Prof **Yibin Wang**
Department of Anesthesiology, Cardiovascular Research Laboratories, David Geffen School of Medicine, University of California, Los Angeles, USA; Tel: 310-206-5197; Email: yibinwang@mednet.ucla.edu

Prof **Hongliang Li**
Department of Cardiology, Zhongnan Hospital of Wuhan University, Institute of Model Animal of Wuhan University, 169 Donghu Road, Wuhan 430071, China; Tel/Fax: 86-27-68759302; Email: lihl@whu.edu.cn

Online Table S1. Laboratory examinations of patients with COVID-19 at admission.

Parameters	With biomarkers (N = 3219)	Without biomarkers (N = 2814)	*P* value
Neutrophil count increase, n/N(%)	447/3199(14.0)	250/2169(11.5)	0.010
Lymphocyte count decrease, n/N(%)	1407/3200(44.0)	639/2169(29.5)	<0.001
CRP increase, n/N(%)	1405/2393(58.7)	406/1068(38.0)	<0.001
AST increase, n/N(%)	696/3112(22.4)	273/1887(14.5)	<0.001
Creatinine increase, n/N(%)	239/2982(8.0)	106/2025(5.2)	<0.001
Glucose increase, n/N(%)	1225/2990(41.0)	531/1855(28.6)	<0.001
D-dimer increase, n/N(%)	1114/2879(38.7)	643/1595(40.3)	0.303
Dyslipidemia, n/N(%)	1481/2521(58.8)	727/1747(41.6)	<0.001
SpO2 < 90%, n/N(%)	117/2103(5.6)	75/2115(3.6)	0.002

CRP, C-reactive protein; AST, aspartate transaminase.

Online Table S2. The absolute or relative values of laboratory examinations in COVID-19 patients with and without myocardial biomarker measurement.

**Parameters**	**With biomarkers (N = 3219)**	**Without biomarkers (N = 2814)**	***P* value**
**Laboratory examinations**, median (IQR)			
Neutrophil count (10^9/L)	3.3(2.4-4.9)	3.4(2.4-4.6)	0.972
CRP (mg/L)	13.2(5-50.6)	5.0(2.2-20.07)	<0.001
AST (U/L)	25.0(18.0-37.25)	21.0(16.0-30.9)	<0.001
D-dimer (relative for ULN)	0.7(0.4-1.72)	0.5(0.1-1.4)	<0.001
Hs-cTnI (relative for ULN)	0.1(0.1-0.3)	-	
CK-MB (relative for ULN)	0.4(0.2-0.5)	-	
BNP or NT-proBNP (relative for ULN)	0.2(0.1-0.4)	0.3(0.1-0.8)	<0.001
CK (relative for ULN)	0.3(0.2-0.6)	0.3(0.2-0.5)	0.727
MYO (relative for ULN)	0.3(0.2-0.6)	0.3(0.2-0.7)	0.510

CRP, C-reactive protein; AST, aspartate transaminase; hs-cTnI, high-sensitivity cardiac troponin I; CK-MB, creatine phosphokinase-MB; CK, creatine phosphokinase; MYO, myoglobin; BNP, brain natriuretic peptide; NT-proBNP, NT-proB-type natriuretic peptide.

Online Table S3. The Normal Range of Serum D-dimer, Hs-cTnI, CK-MB, BNP, NT-proBNP, CK, MYO Test in Study Hospitals

Site	D-dimer (mg/L)	Hs-cTnI (ng/mL)	CK-MB	BNP (pg/mL)	NT-proBNP (pg/mL)	CK (U/L)	MYO (ng/mL)
1	0-0.5	0-26.2	0-25(U/L)	0-100	0-450/ 0-900/ 0-1800	0-171/ 0-145	0-140.1
2	0-0.55	0-0.04	0-5(ng/mL)	-	0-450/ 0-900/ 0-1800	50-310/ 40-200	0-110
3	0-0.5	0-0.04/ 0-0.03	0-25(U/L)	2-121	-	30-180	0-110
4	0-1	0-0.1/ 0-0.4	0-5.9(ng/mL)/ 0-25(U/L)	-	0-900	50-310/ 40-200	0-100
5	0-1	0-0.09/ 0-0.03/ 0-0.0156	0-25(U/L)/ 0-24(U/L)	0-100/ 0-125	-	26-140/ 38-174/ 0-170/ 0-190	17.4-105.5/ 14.3-65.8
6	0-0.243	0-0.1	0-6.22(ng/mL)	-	0-222	50-310/ 40-200	7.4-105.7
7	0-0.55	0-0.04	0-4.97(ng/mL)/ 0-6.36(ng/mL)	0-100	-	0-145/ 0-171	0-80/ 0-65
8	0-0.55/ 0-1	0-0.026	0-24(U/L)		0-300/ 0-450/ 0-900/ 0-1800	30-120	0-154.9
9	0-0.243	0-0.5	0-25(U/L)/ 0.5-6.3(ng/mL)	0-100	0-300	0-190	1.5-70/ 0-100

Online Table S4. Akaike's Information Criterion (AIC) of univariate logistic regression models

**Variables**	**AIC**	**-2log-likelihood**	***P* value**
Hs-cTnI increase	634.780	-315.390	<0.001
(NT-Pro)BNP increase	846.171	-421.086	<0.001
MYO increase	919.554	-457.777	<0.001
SpO2 < 95%	1088.586	-542.293	<0.001
CK increase	1141.665	-568.832	<0.001
Neutrophil count increase	1287.439	-641.719	<0.001
CRP increase	1289.833	-642.917	<0.001
CK-MB increase	1306.155	-651.078	<0.001
D-dimer increase	1309.915	-652.958	<0.001
Lymphocyte count decrease	1332.133	-664.067	<0.001
Age	1342.802	-669.401	<0.001
AST increase	1346.803	-671.402	<0.001
Creatinine increase	1375.480	-685.740	<0.001
With hypertension	1425.938	-710.969	<0.001
Gender	1433.874	-714.937	<0.001
With coronary heart disease	1440.715	-718.357	<0.001
With cerebrovascular disease	1449.493	-722.747	<0.001
With diabetes	1453.164	-724.582	<0.001
Fatigue	1463.075	-729.538	0.238
Fever	1463.399	-729.700	0.313
SBP	1463.598	-729.799	0.354
With COPD	1464.118	-730.059	0.543
DBP	1464.222	-730.111	0.637

SBP, Systolic blood pressure; DBP, Diastolic blood pressure; COPD, Chronic obstructive pulmonary disease; AST, aspartate transaminase; hs-cTnI, high-sensitivity cardiac troponin I; CK-MB, creatine phosphokinase-MB; CK, creatine phosphokinase; MYO, myoglobin; BNP, brain natriuretic peptide; NT-proBNP, NT-proB-type natriuretic peptide.

Online Table S5. The outcomes in patients with and without heart injury marker measured

**Outcomes**	**With biomarkers (N = 3219)**	**Without biomarkers (N = 2814)**	***P* value**
All-cause death	193/3219(6.0%)	107/2814(3.8%)	<0.001
ARDS	430/3219(13.4%)	207/2814(7.4%)	<0.001
Heart failure	331/3219(10.3%)	184/2814(6.5%)	<0.001
DIC	25/3219(0.8%)	8/2814(0.3%)	0.016
Sepsis/MOF	102/3219(3.2%)	30/2814(1.1%)	<0.001
Acute renal failure	96/3219(3.0%)	38/2814(1.4%)	<0.001

ARDS, acute respiratory distress; DIC, disseminated inravascular coagulation; MOF, multi-organ failure.

Online Table S6. The causes of death in patients with and without heart injury

Causes	With heart injury (N = 126)	Without heart injury (N = 75)	*P* value
Respiratory failure, n/N(%)	115 (91.3)	72 (96.0)	0.323
MODS, n/N(%)	15 (11.9)	5 (6.7)	0.339
DIC, n/N(%)	10 (7.9)	2 (2.7)	0.223
Cardiac arrest, n/N(%)	7 (5.6)	4 (5.3)	1.000
Hyperkalemia, n/N(%)	7 (5.6)	1 (1.3)	0.268
Heart failure , n/N(%)	6 (4.8)	2 (2.7)	0.717
Sepsis, n/N(%)	3 (2.4)	1 (1.3)	1.000
Malignant arrhythmia, n/N(%)	3 (2.4)	1 (1.3)	1.000

MODS, multiple organ dysfunction syndrome; DIC, disseminated intravascular coagulation.

Online Table S7. Overall performance of increased myocardial marker levels above ULNs for predicting COVID-19 mortality

Parameters	Hs-cTnI	CK-MB	(NT-Pro)BNP	CK	MYO
N	1462	3120	1650	2534	1895
Mortality	98	178	133	163	148
AUC(95% CI)	0.78 (0.73-0.84)	0.71 (0.67-0.75)	0.81 (0.78-0.85)	0.67 (0.62-0.72)	0.83 (0.80-0.86)
Cutoff (relative to ULN)	1	1	1	1	1
Accuracy, % (95% CI)	91.72 (90.19,93.09)	91.25 (90.20,92.22)	85.94 (84.17,87.58)	86.03 (84.62,87.36)	87.44 (85.86,88.90)
Sensitivity, %	36.74	21.91	42.86	37.42	46.62
Specificity, %	95.67	95.45	89.72	89.37	90.90
PPV	0.38	0.23	0.27	0.19	0.30
NPV	0.95	0.95	0.95	0.95	0.95
Balanced accuracy	0.66	0.59	0.66	0.63	0.69

AUC, area under the ROC curves; PPV, positive predictive value; NPV, negative predictive value; hs-cTnI, high-sensitivity cardiac troponin I; CK-MB, creatine phosphokinase-MB; CK, creatine phosphokinase; MYO, myoglobin; BNP, brain natriuretic peptide; NT-proBNP, NT-proB-type natriuretic peptide.

Online Table S8. Associations of cardiac injury markers above cutoffs with 28-day all-cause mortality of COVID-19 in patients divided by cutoffs and upper limit of normal

	Crude		Model I		Model II	
	HR(95% CI)	*P* value	HR(95% CI)	*P* value	HR(95% CI)	*P* value
Hs-cTnI	13.39(8.88, 20.20)	＜0.001	10.68(6.87, 16.59)	＜0.001	4.74(3.05,7.35)	＜0.001
CK-MB	3.48(2.58, 4.71)	＜0.001	3.63(2.60, 5.06)	＜0.001	2.17(1.56,3.01)	＜0.001
(NT-Pro)BNP	14.33(7.73, 26.56)	＜0.001	12.01(6.39, 22.58)	＜0.001	5.67(2.97,10.82)	＜0.001
CK	2.91(2.11, 4.01)	＜0.001	2.70(1.92, 3.81)	＜0.001	1.80(1.28,2.53)	＜0.001
Myoglobin	7.32(5.07, 10.59)	＜0.001	5.00(3.37, 7.42)	＜0.001	2.74(1.82,4.13)	＜0.001
CRP	7.73(4.91,12.16)	＜0.001	6.63(4.08, 10.76)	＜0.001	1.65(0.98,2.77)	0.059
D-dimer	8.22(5.96-11.34)	＜0.001	6.40(4.55, 9.01)	＜0.001	5.55(3.32,9.30)	＜0.001

Model I: Adjusted for age, sex, co-existing diseases (diabetes, hypertension, coronary heart disease, and cerebrovascular disease), hospital sites (as a random effect).

Model II: Adjusted for age, sex, co-existing diseases (diabetes, hypertension, coronary heart disease, and cerebrovascular disease) and indicators of disease severity (CRP increase, Neutrophil count increase, Lymphocyte count decrease, D-dimer increase and SpO2<95%), hospital sites (as a random effect).

hs-cTnI, high-sensitivity cardiac troponin I; CK-MB, creatine phosphokinase-MB; CK, creatine phosphokinase; MYO, myoglobin; BNP, brain natriuretic peptide; NT-proBNP, NT-proB-type natriuretic peptide.

Online Table S9. Akaike's Information Criterion (AIC) of Mixed-effect Cox models

Variables	AIC		
	Model I[a]	Model II[b]	Model III[c]
Hs-cTnI	168.110	160.932	265.221
CK-MB	218.818	221.221	502.337
(NT-Pro)BNP	170.389	167.363	341.531
CK	142.939	144.169	414.688
Myoglobin	171.863	169.988	352.756

hs-cTnI, high-sensitivity cardiac troponin I; CK-MB, creatine phosphokinase-MB; CK, creatine phosphokinase; MYO, myoglobin; BNP, brain natriuretic peptide; NT-proBNP, NT-proB-type natriuretic peptide.

a. Adjusted for age, sex, hospital sites (as a random effect).

b. Adjusted for age, sex, hospital sites (as a random effect), co-existing diseases (diabetes, hypertension, coronary heart disease, and cerebrovascular disease).

c. Adjusted for age, sex, hospital sites (as a random effect), co-existing diseases (diabetes, hypertension, coronary heart disease, and cerebrovascular disease) and indicators of disease severity (CRP increase,Neutrophil count increase, Lymphocyte count decrease, D-dimer increase and SpO_2<95%).

Online Table S10. The interaction associations of cardiac injury markers and inflammatory factors with 28-day all-cause of death in patients with COVID-19

	CRP ≤ Cut-off	CRP > Cut-off		D-dimer ≤ Cut-off	D-dimer > Cut-off	
	aHR[a] （95%CI）	aHR[a] （95%CI）	Interaction *P* value	aHR[a] （95%CI）	aHR[a] （95%CI）	Interaction *P* value
CK-MB ≤ Cut-off	Ref	6.24 (3.17,12.28)		Ref	3.82 (2.27,6.43)	
CK-MB > Cut-off	4.44 (1.90,10.35)	21.39 (10.81,42.32)	0.588	2.18 (1.22,3.92)	18.12 (11.09,29.58)	0.029
CK ≤ Cut-off	Ref	11.92 (5.22,27.24)		Ref	7.34 (3.91,13.78)	
CK > Cut-off	6.20 (2.48,15.53)	18.16 (8.00,41.19)	0.006	3.84 (2.04,7.22)	20.10 (10.98,36.78)	0.377
MYO ≤ Cut-off	Ref	13.82 (4.64,41.13)		Ref	5.62 (2.84,11.15)	
MYO > Cut-off	16.06 (5.32,48.44)	41.36 (14.48,118.14)	0.007	4.25 (2.16,8.37)	23.84 (12.89,44.10)	0.996
Hs-cTnI ≤ Cut-off	Ref	4.96 (2.27,10.85)		Ref	10.58 (4.78,23.42)	
Hs-cTnI > Cut-off	15.76 (5.83,42.60)	37.40 (17.15,81.54)	0.190	14.71 (6.09,35.52)	83.72 (38.41,182.49)	0.222
(NT-Pro)BNP ≤ Cut-off	Ref	3.51 (1.01,12.21)		Ref	2.95 (0.79,11.04)	
(NT-Pro)BNP > Cut-off	6.63 (2.13,20.64)	28.87 (10.26,81.24)	0.759	6.26 (2.33,16.83)	43.96 (17.49,110.46)	0.226

a. Adjusted for age, sex, hospital sites (as a random effect), co-existing diseases (diabetes, hypertension, coronary heart disease, and cerebrovascular disease).

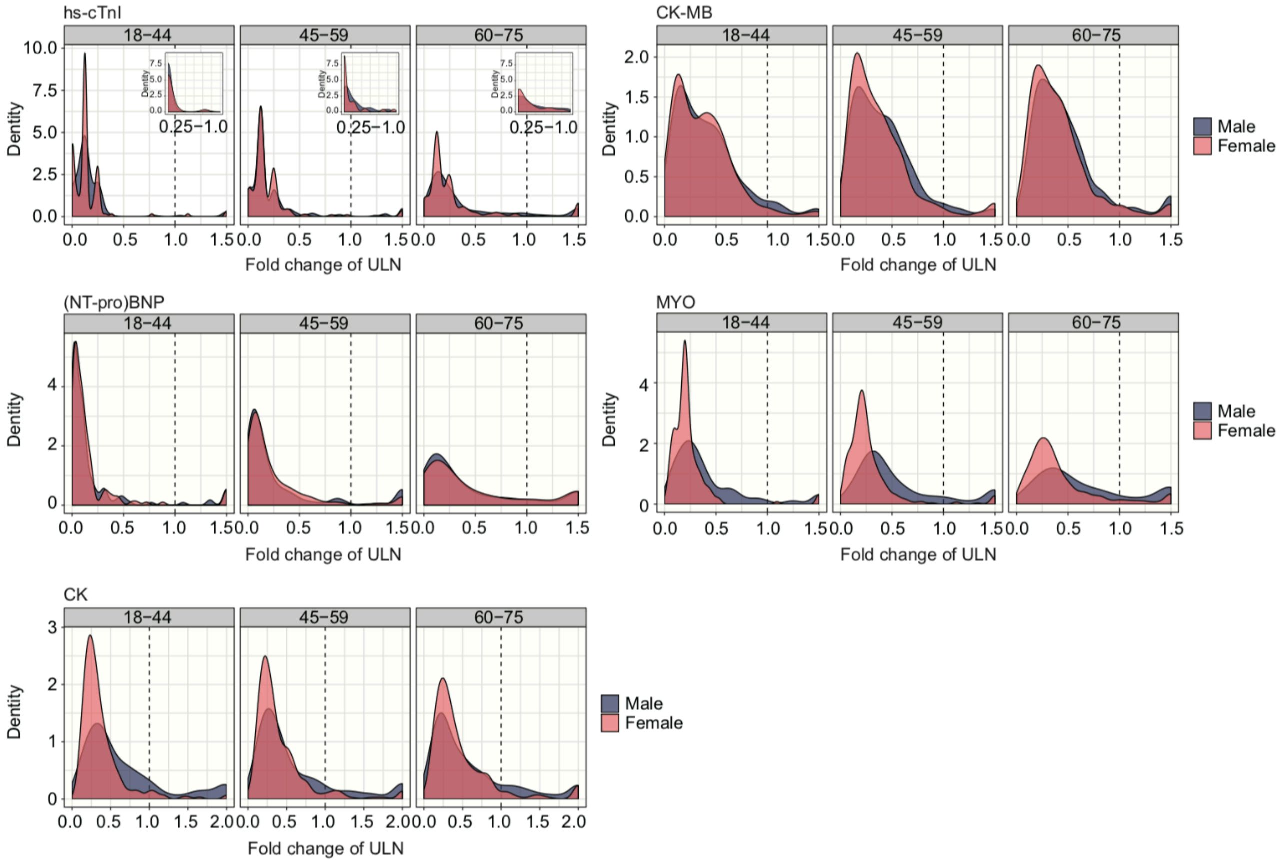

Online Figure S1. The distributions of serum hs-cTnI, CK-MB, (NT-pro)BNP, CK, and MYO on admission at the age brackets of 18-44, 45-59, and 60-75 years among patients with COVID-19. The x-axis represents relative contents of myocardial markers levels to their corresponding ULNs according to criteria in each hospital.

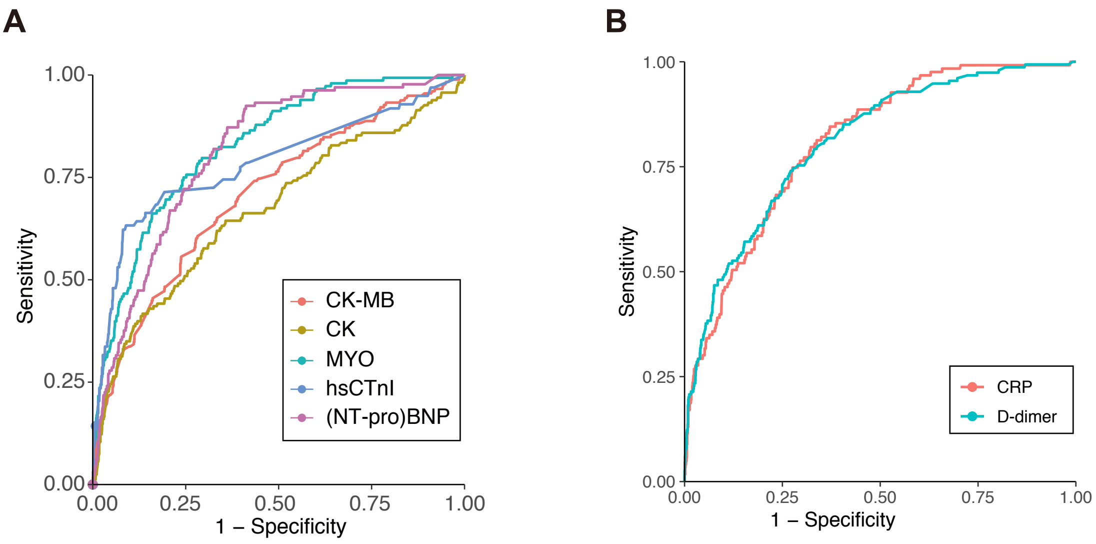

Online Figure S2. ROC curve showing overall performance of myocardial injury markers and inflammatory factors for identifying risk of COVID-19 mortality.

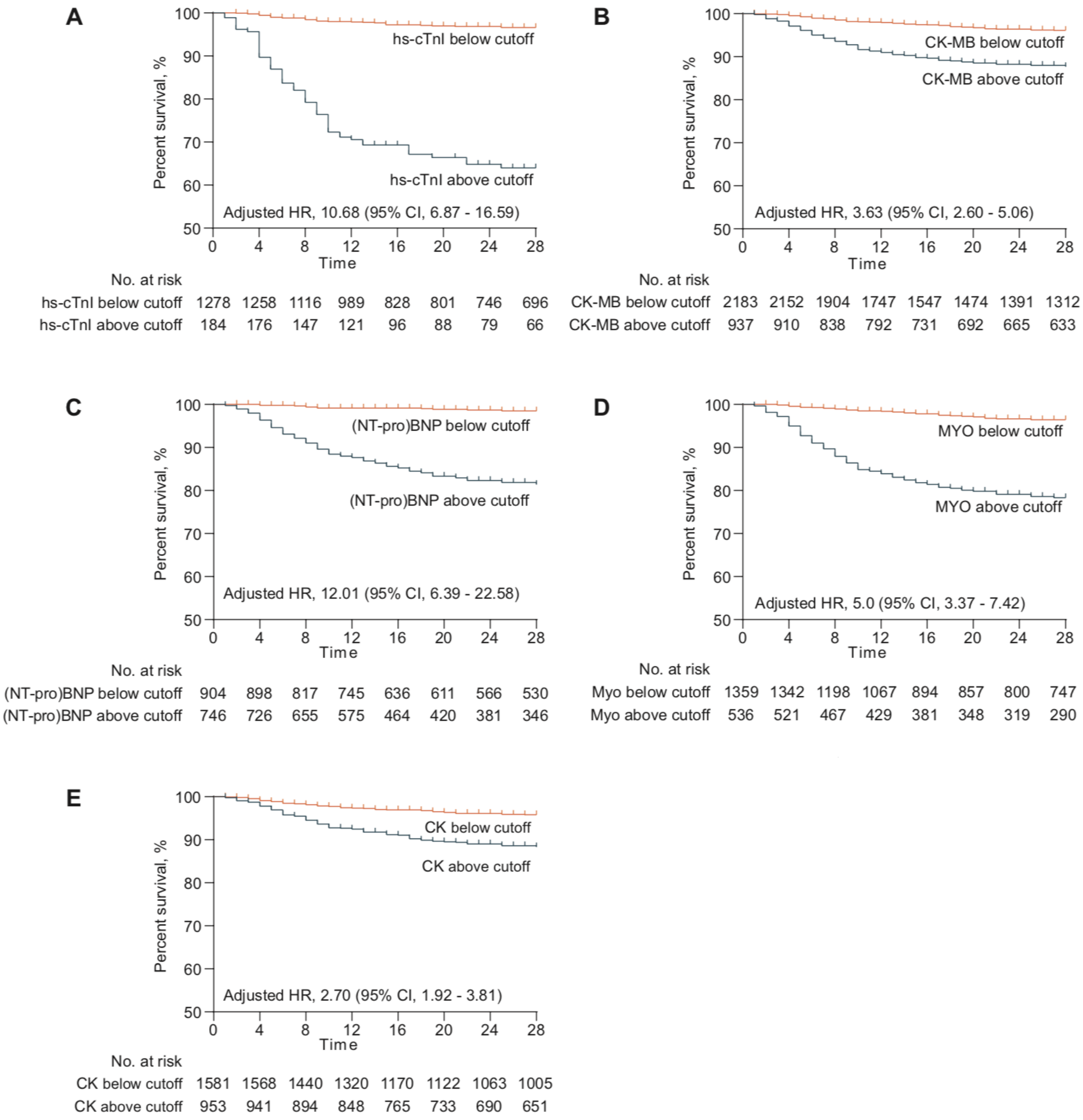

Online Figure S3. Kaplan-Meier Curves showing cumulative survival of COVID-19 among patients with levels of hs-cTnI (A), CK-MB (B), (NT-pro)BNP (C), MYO (D), and CK (E) above and under cutoffs.

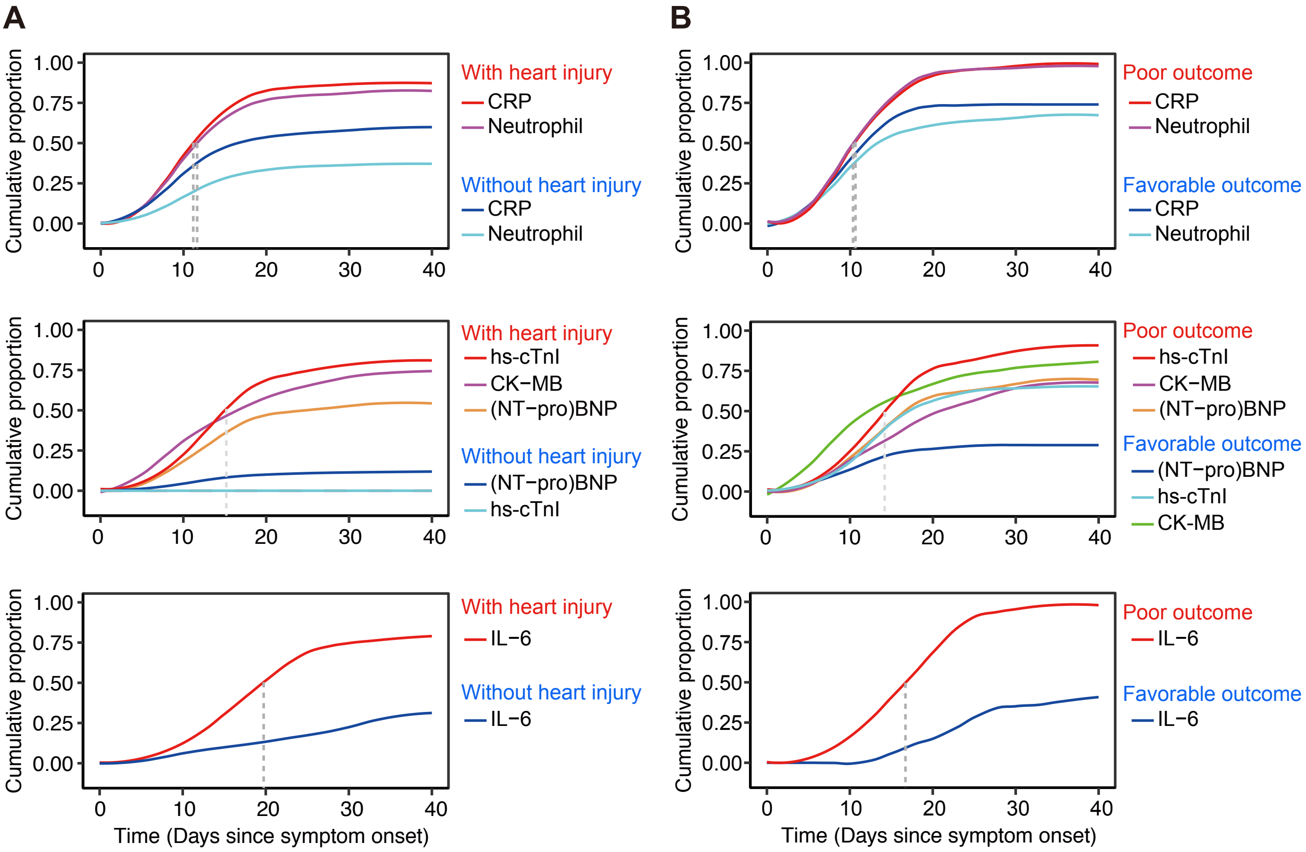

Online Figure S4. Trajectory and levels of cardiac biomarkers and inflammatory markers among COVID-19 patients with or without heart injury (A) and among patients with heart injury and having favorable or poor outcomes (B).

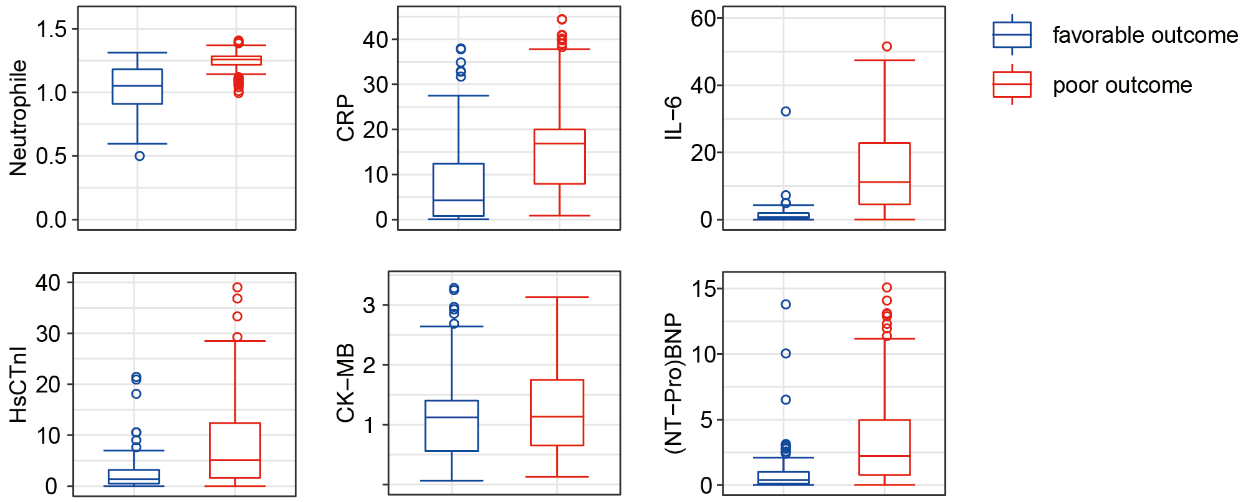

Online Figure S5. The peak values of serum cardiac biomarkers and inflammatory factors levels relative to their ULNs during hospitalization in patients with favorable or poor outcomes.
