# Supplementary material for: Redefining Cardiac Biomarkers in Predicting Mortality of Inpatients With COVID-19
Source: Hypertension. 2020 Jul 14;76(4):1104–12. doi: 10.1161/HYPERTENSIONAHA.120.15528 (PMC7375179; doi:10.1161/HYPERTENSIONAHA.120.15528)
Supplement: Supplementary file 2 [file hyp-76-1104-s002.docx]

**Tweet.**

Cardiac injury markers showed high performance to predict the risk of COVID-19 mortality, but with actually much lower cutoffs than standard range, being about 50% lower.
