# Supplementary figures and images for: Redefining Cardiac Biomarkers in Predicting Mortality of Inpatients With COVID-19
Source: Hypertension. 2020 Jul 14;76(4):1104–12. doi: 10.1161/HYPERTENSIONAHA.120.15528 (PMC7375179; doi:10.1161/HYPERTENSIONAHA.120.15528)

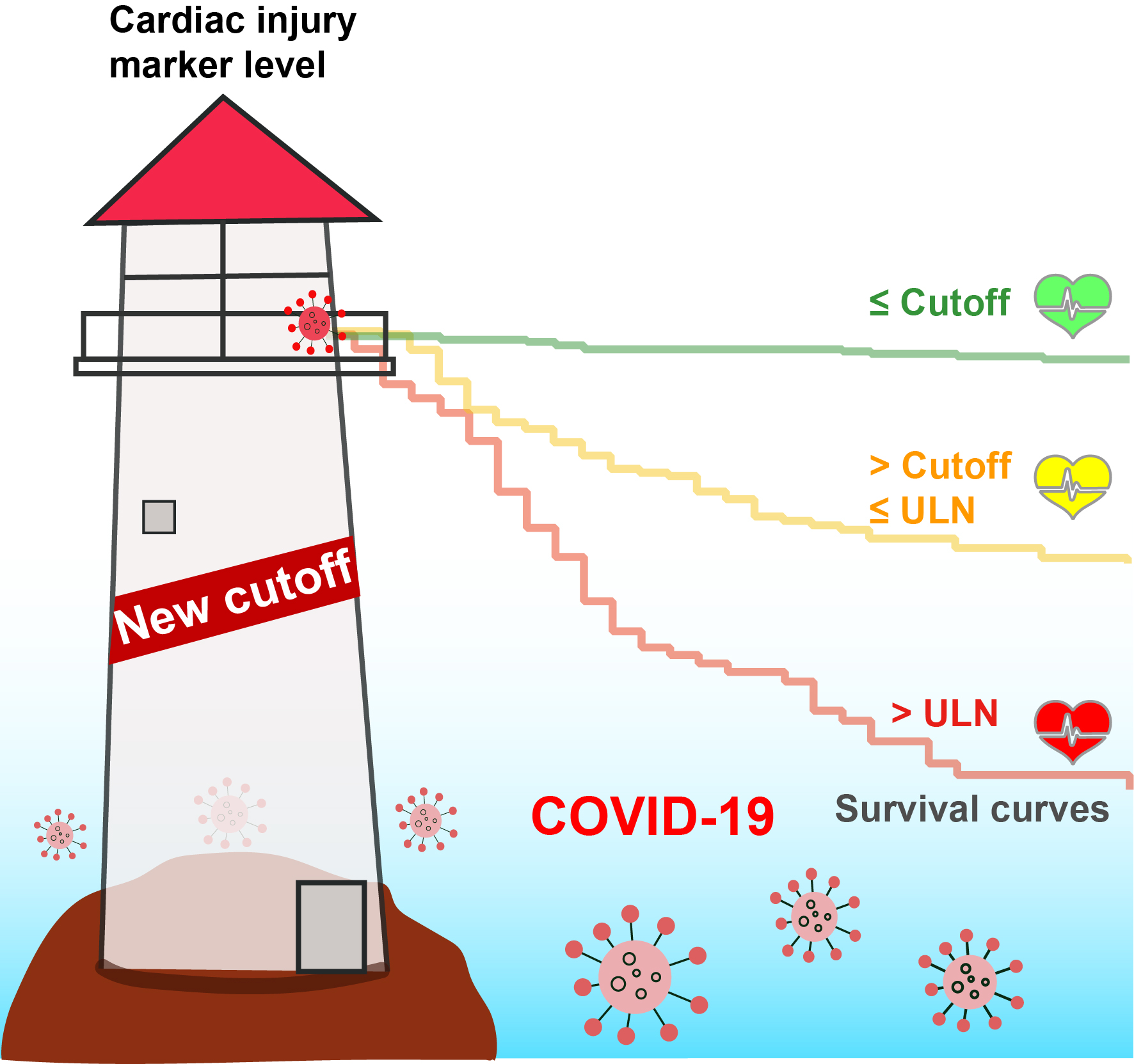

Supplement: Supplementary file 3 [file hyp-76-1104-s003.jpg]
